# Enhancing Bystander Intervention: Insights from the Utstein Analysis of Out-of-Hospital Cardiac Arrests in Slovenia

**DOI:** 10.3390/medicina60081227

**Published:** 2024-07-29

**Authors:** Luka Petravić, Rok Miklič, Evgenija Burger, Urša Keše, Domen Kulovec, Eva Poljanšek, Gašper Tomšič, Tilen Pintarič, Miguel Faria Lopes, Miha Brezovnik, Matej Strnad

**Affiliations:** 1 Emergency Care Department, University Medical Center Maribor, Ljubljanska ulica 5, 2000 Maribor, Slovenia; strnad.matej78@gmail.com; 2 Faculty of Medicine, University of Maribor, Taborska ulica 8, 2000 Maribor, Slovenia; rok.miklic@student.um.si; 3 Faculty of Mathematics and Physics, University of Ljubljana, Jadranska ulica 21, 1000 Ljubljana, Slovenia; burger.evgenija@gmail.com; 4 Faculty of Computer and Information Science, University of Ljubljana, Večna pot 113, 1000 Ljubljana, Slovenia; ursa.kese@gmail.com; 5 General Hospital Novo mesto, Šmihelska cesta 1, 8000 Novo mesto, Slovenia; domen.kulovec@outlook.com; 6 Sava med, d.o.o., Cesta k Dravi 8, 2241 Spodnji Duplek, Slovenia; eva.poljansek@gmail.com; 7 Faculty of Pharmacy, University of Ljubljana, Aškerčeva cesta 7, 1000 Ljubljana, Slovenia; gasper.tomsic@ffa.uni-lj.si; 8 Faculty of Mechanical Engineering, University of Novo mesto, Na Loko 2, 8000 Novo mesto, Slovenia; tilen.pintaric99@gmail.com; 9 Faculty of Electrical Engineering and Computer Science, University of Maribor, Koroška cesta 46, 2000 Maribor, Slovenia; miguelfarialopes2000@gmail.com; 10 Medical Emergency Dispatch Center, University Medical Center Ljubljana, Trg Leona Štuklja 10, 2000 Maribor, Slovenia; miha.brezovnik@kclj.si; 11 Prehospital Unit, Emergency Medical Services, Community Healthcare Center Maribor, Ul. talcev 9, 2000 Maribor, Slovenia

**Keywords:** out-of-hospital cardiac arrest, return of spontaneous circulation, Slovenia, education, heart arrest, public health, emergency medical services, emergency medical dispatch

## Abstract

*Background and Objectives*: Out-of-hospital cardiac arrest (OHCA) and survival is a pressing matter all around the world. Despite years of research and great strides and advancements, survival remains alarmingly low. The aim of this study was to measure the survival and characteristics of patients having an OHCA in Slovenia, with an in-depth look at how the bystanders affect the return of spontaneous circulation (ROSC) and survival of OHCA. *Materials and Methods*: In this observational cross-sectional prospective study, we enrolled patients between 1 September 2022 and 30 November 2022, with a follow-up period of 1 month. All OHCAs attended by the emergency medical services were included. Data were collected and analyzed according to the Utstein 2015 reporting template. Independent predictors of ROSC and 30-day survival or survival were explored using ridge regression. *Results*: ROSC was achieved in 41% of cases where resuscitation was attempted. The overall 30-day survival rate where resuscitation was attempted was 14%. In 13% of all cases where resuscitation was attempted, patients had a favorable neurological outcome. Using our prediction model, we found that defibrillation under 20 min and ventricular fibrillation as an initial rhythm improves survival, whilst no defibrillation and bystander full cardiopulmonary resuscitation negatively predicted survival. *Conclusions*: Slovenia has OHCA 30-day survival comparable to the rest of the European Union. The favorable neurological outcome is high. Our data show that bystanders do not significantly improve survival. This represents an untapped potential of general public education in cardiopulmonary resuscitation and automatic external defibrillator use. Following good practices from abroad and improving layperson CPR knowledge could further improve OHCA survival.

## 1. Introduction

Out-of-hospital cardiac arrest (OHCA) is a medical emergency characterized by the absence of mechanical heart action and systemic circulation outside a hospital environment [1]. The annual incidence of OHCA in Europe and the United States of America has historically been documented at 275,000 and 420,000 cases, respectively [2,3].

Despite extensive efforts to improve the management of OHCA, survival rates remain alarmingly low [4]. Most patients die before being admitted to the hospital. According to estimates, only 7.7% of patients with OHCA will survive globally [4].

Coordination of the “chain of survival” is essential for successful outcomes [3,5]. It is imperative to recognize the patient having an OHCA and for bystanders to start performing good-quality cardiopulmonary resuscitation (CPR) as soon as possible [6]. In Slovenia, studies on bystander CPR are scarce. Slovenian bystanders are still reluctant to initiate CPR: between 2008 and 2012, only 50.6% of bystanders began CPR prior to EMS arrival, and only 36.5% of them performed CPR correctly [7]. This is still low compared to some other countries on the European continent, such as Norway (rate of 85%) and Czech Republic (rate of 84%) [8].

The vast majority of European countries have yet to institute mandatory CPR training in school curricula. Prevailing obstacles include low political motivation, concerns about the cost and availability of automatic external defibrillators (AEDs), and the perception that teachers might be overburdened by the addition of CPR courses [9].

The aim of this observational study was to measure survival and characteristics of patients having an OHCA in Slovenia, with a deep look at the bystander effect on the return of spontaneous circulation (ROSC) and survival of OHCA patients.

## 2. Materials and Methods

### 2.1. Study Design

In this observational, cross-sectional, multicenter prospective study, we enrolled all OHCAs attended by EMS between 1 September and 30 November 2022 with a follow-up period of 1 month, ending data collection on 31 December 2022. Data were collected and analyzed according to the Utstein 2015 reporting template [10]. The study adhered to the Strengthening the Reporting of Observational Studies in Epidemiology (STROBE) statement [11]. This study was approved by an ethical review board of the Republic of Slovenia (0120-153/2022/3).

### 2.2. Study Area and Population

The study took place in various parts of Slovenia and included 18 different EMSs. The catchment area of the study was inhabited by 1,520,895 persons, covering an area of 10,404 km^2^, and the GDP of Slovenia in 2022 was USD 28,473 per capita [12]. The study population was composed of anyone having an OHCA, regardless of age or OHCA etiology. Slovenian citizens mandatorily train in first aid only once in their lives—when applying to get a driver’s permit. Learning CPR is not mandatory in grade or high school. If a citizen wants to revise their knowledge, he or she must do it on a voluntary basis, frequently in exchange for payment.

### 2.3. Description of the Emergency Medical System

The system has undergone some changes since being reported on between 2001 and 2004 by Grmec et al. [13], with most of the reforms still in progress and unfinished. There is a single 112 emergency number. After calling 112, the caller is directed to the Regional Notification Centre (ReNC), where a telephone operator decides what emergency services are needed. In Slovenia, there are currently two concurrent systems of dispatch. Some geographical areas are covered by the medical dispatch center, which handles the logistics and activates the EMS (new system), while other parts are in the process of implementing the medical dispatch system, but without the actual coverage of dispatchers, the call is redirected directly to the local EMS, which handles logistics and activates itself (old system). The old system entails an emergency doctor picking up the phone, organizing the drive out, going to the field and giving the instructions to the caller—if he manages to. The dispatch center has a two-person system in place, one receiving the call and one dispatching the necessary teams to the scene at the same time. The dispatchers are using Norsk indeks for medisinsk nødhjelp 2017 translated into Slovenian and work only on manning the phone and organizing the logistics of each call [14]. An overview of the system is available in Supplementary Figure S1.

Care for emergencies in the out-of-hospital setting is provided by advanced life support (ALS) teams and basic life support (BLS) teams. Each ALS team includes an emergency medicine-trained physician providing ALS, and BLS teams comprise a registered nurse and/or medical technician. Upon receiving an emergency call with suspicion of OHCA, the ALS team is dispatched to the scene. If OHCA is not suspected and the patient does not need immediate critical care, a BLS team is dispatched. Where the ALS team is busy with another case, the BLS team is dispatched and the ALS team joins later [15]. In remote areas, first responders are dispatched to shorten the time between the arrest and BLS [16]. BLS and ALS are provided according to the current European Resuscitation Council guidelines and clinical algorithms [17].

### 2.4. Data Collection

Data collection was carried out using a paper form using Utstein 2015 variables [10]. The data were then manually entered into custom software created by the authors and aggregated. A translation of the form for data collection is available with the data file [18]. To address potential bias, there were no free-text variables. Each variable had a clear definition as per Utstein 2015. There was no case selection, as each patient having an OHCA was in the catchment area and entered into the database. The study size was not calculated in advance. The end point was defined with a point in time.

### 2.5. Statistical Methodology

Continuous data are reported on with means and confidence intervals of 95%. Comparison of nominal values used the χ^2^ test and Fisher’s exact test. Comparisons between groups were made using *t*-tests or Mann–Whitney-U test when exploring differences between distributions.

The result was considered significant when the p value was under 0.05. Independent predictors of ROSC and survival were explored using multinomial regression. The analysis was conducted using IBM SPSS Statistics (v28.0.1.1 (14)).

### 2.6. Model Development

Independent predictors of ROSC and survival were explored using lasso regression. Target variables were ROSC (possible outcome being ROSC, no ROSC) and survival till hospital discharge or 30-day survival (possible outcomes being yes, no/unknown). Classes of target variables were encoded as integers.

The features chosen for our model were the core Utstein features. Features that were not relevant to ROSC or survival were eliminated. Incidence was calculated using methodology described by Tenny and Boktor [19].

Data preprocessing was minimal. Missing values for categorical features were encoded as unknown. In cases of numerical variables, there were missing values for response times and defibrillation times.

We aimed to develop a logistic regression model, which was chosen due to its popularity in medical research and due to its ability to study the relationship between binary outcomes and given features. We also developed a random forest model and studied feature importance due to its robustness and general acceptance. We evaluated our models by computing the mean log score after leave-one-out cross-validation. We also report mean model accuracy. The code used is openly available [20]. Additional methods are available as supplemental material accompanying the online article.

## 3. Results

The Utstein-style results are presented below (Table 1). From the total number of cases and population, we calculated the yearly incidence of OHCA in Slovenia to be 77.32 per 100,000 inhabitants. Extrapolating to the Slovenian population of 2,120,937, there is a yearly case volume of 1639 patients for the whole of Slovenia [21]. Out of 277 patients, 23% were not able to perform all activities of daily living without the assistance of caregivers. Out of 239 patients, 90% had a documented history of other disease conditions that existed before the cardiac arrest.

Comparing return of spontaneous circulation (ROSC) and 30-day survival rates in cases where first responders were activated did not yield statistically significant results. Both analyses demonstrated low statistical power due to the small sample (Pearson’s χ^2^ test: for ROSC, *p* = 0.32, power = 0.17; for 30-day survival, *p* = 0.288, power = 0.19).

Subsets of patients were further divided depending on bystander response (Table 2). EMSs performed CPR in most of the cases (85.5%) where the dispatcher provided CPR instructions. Where the dispatcher did not provide CPR instructions, none of the patients was resuscitated by the EMS team.

We hypothesized that a bystander performing CPR with both ventilation and chest compressions (CCs), only CCs, or no CPR is independent of the dispatcher giving CPR instructions, and this hypothesis was rejected (*p* < 0.001). In 144 (70%) out of 206 cases where CPR instructions were given, the bystander performed CPR. Only in 5 (9%) out of 53 cases where CPR instructions were not given did the bystander perform CPR. Out of 53 cases where CPR instructions were not given by the dispatcher, the cardiac arrest was not recognized by the dispatcher in 11 cases (21%), in 23 cases (43%), it is unknown whether it was recognized or not, and in 19 cases (36%), the cardiac arrest was recognized by the dispatcher.

We found that shockable rhythms had significantly higher chances of achieving ROSC than non-shockable (*p* = 0.00027).

Asystole was the most common first rhythm in the home setting (49.2%), public buildings (40.0%), and recreational facilities (50%). VF was most common on the roads (32.4%) and at work (100%). PEA was the most common first rhythm in nursing homes (31.3%).

### 3.1. Bystanders

#### Bystander Response and Location of OHCA

We have found that the highest bystander CPR rate was at work, sports or recreational facilities, and public buildings (100%), but those are also the places with the lowest absolute number of OHCAs, accounting for only 3% of all the OHCAs in our sample. OHCA mostly happens in a home setting (69%), where the CPR rate is 74.2%. The CPR rate is higher for nursing homes (81.3%). A notable observation revealed a high CPR rate of 94.0% at the roadside.

Comparing survival of those who received bystander CPR and of those who did not, we found no significant difference (*p* = 0.24).

OHCA was witnessed by a bystander 88% of the time in nursing homes, 74% on the road, and 60% in public buildings. Bystanders witnessed an OHCA in 55% and 50% of cases at home and sports or recreational facilities, respectively.

The technique used for CPR significantly differed, with at-home bystander CPR consisting of both rescue breaths and chest compressions in 4% of cases and chest compressions only in 42.9%. The highest rate of full CPR was in nursing homes—31.3% of cases. The rate of chest compressions only was the highest on the roads—58.8% of all cases.

Using regression, we found that the R^2^ values for bystander CPR were negative (Table 3) for ROSC, 30-day survival, and survival until discharge.

### 3.2. Results—Logistic Regression

We built a logistic regression model to determine the effects of variables on outcome (ROSC or survival). Mean log score after leave-one-out cross-validation of the model where the target variable was ROSC was 0.544, and for the model where the target variable was survival until discharge or 30-day survival was 0.155. A lower log loss suggests that the model’s predicted probabilities are closer to the actual outcomes, although in our dataset, the target variable describing survival was very imbalanced: only 27 (13%) cases ended in survival, while 179 (87%) cases ended in death or unknown outcome. This can affect the model such that it is biased towards the majority class and has lower sensitivity.

### 3.3. Predicting ROSC

**Figure 1:**
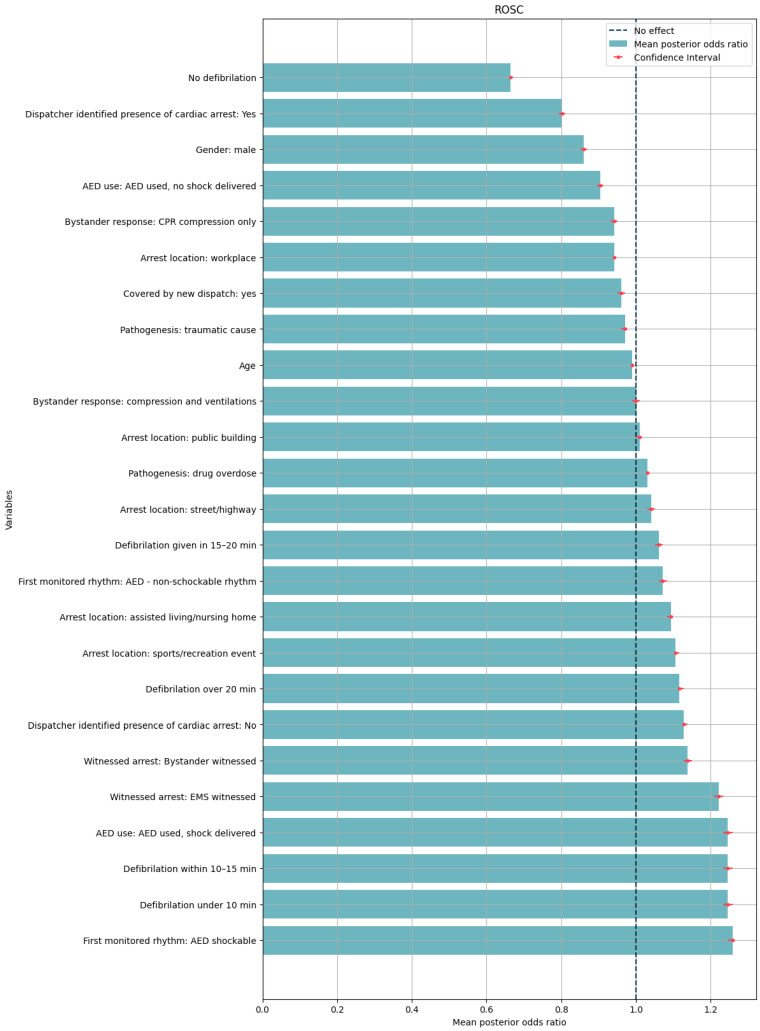
Mean posterior odds ratio for selected variables in predicting ROSC (left of no-effect line: worsens, right of no-effect line: improves, confidence interval 90th percentile, ROSC: return of spontaneous circulation).

For bystander response (Figure 1), we observed that the odds of ROSC increase if the event was witnessed by a bystander compared to the event of unwitnessed cardiac arrest, but not as much as they increase if the event was witnessed by the EMS. The feature that is connected to the bystander and increases the odds of ROSC the most is the use of AED by a bystander in comparison to AED not being used.

Results from our study also suggest that defibrillation in the first 15 min increases the odds of ROSC (Supplementary Table S1). The event of no defibrillation decreases the odds compared to defibrillation happening and is one of the variables that has the most negative odds.

We found no significant effect on odds concerning response time. However, we observed a negative association between increasing age and the odds of ROSC.

Our model achieved high recall (95%), indicating that it effectively identified a significant portion of the actual positive instances. Additionally, the model exhibited good precision (63%), signifying that among the instances predicted as positive, a large proportion of them are true positives. In other words, the model successfully captures a substantial number of positive instances while maintaining a high level of correctness in its positive predictions. The mean log score of the model was 0.67 and mean accuracy was 65.5%.

### 3.4. Predicting Survival until Hospital Discharge or 30-Day Survival

**Figure 2:**
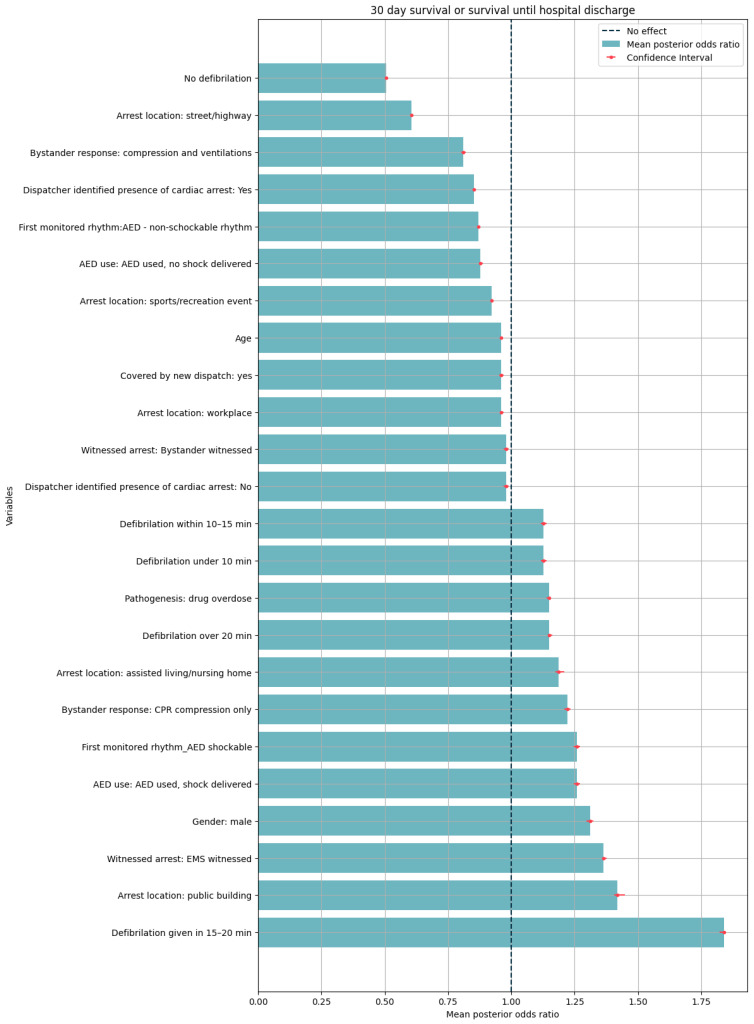
Mean posterior odds ratio for selected variables in predicting survival, both 30 day and until hospital discharge (left of no-effect line: worsens, right of no-effect line: improves, confidence interval 90th percentile).

Similarly, our study findings suggest that defibrillation increases the odds of survival, while the absence of defibrillation decreases them. Interestingly (Figure 2), we observed that the variable indicating defibrillation between the 15th and 20th minute demonstrates the most substantial increase in odds (Supplementary Table S1).

Examining bystander response, our analysis reveals that in cases of survival, bystander CPR increases the odds compared to scenarios with no bystander CPR. However, when bystander CPR is combined with ventilation, we observed a decrease in the odds of survival.

Bystander AED use during defibrillation increases positive outcomes, but when defibrillation does not occur, bystander AED use decreases the odds compared to situations without AED use.

In terms of location, our findings indicate that events occurring in public buildings and nursing homes have higher odds of survival compared to those happening at home.

The model had 94% accuracy and zero false-negative predictions.

## 4. Discussion

This study investigated OHCA outcomes in Slovenia, revealing favorable survival rates. Despite over half of OHCAs being witnessed, bystander involvement showed minimal influence on both ROSC and overall patient outcomes. This finding warrants further exploration to understand the underlying factors influencing bystander intervention and its impact on OHCA survival.

The annual incidence of OHCA in Slovenia was estimated at 77.32 per 100,000 population. This figure is notably lower than the previously calculated rate under Utstein-style guidelines, reported as 114.3 per 100,000 by Tadel et al. in 1998 [22]. Examining the historical context and comparing other ex-Yugoslavian countries, the incidence of OHCA in Slovenia appears relatively lower, with incidence in Croatia, Bosnia and Herzegovina, and Serbia ranging between 62 and 167.9/100,000 [23,24,25]. In 2020, Slovenia reported lower age-standardized death rates from cardiovascular diseases compared to the European average, particularly for males. Among Slovenian men, the rate was 191.0 per 100,000 inhabitants, significantly below the European average of 217.0. For Slovenian women, the rate stood at 138.3 per 100,000, nearly identical to the European average of 138.2 [26].

ROSC was achieved in 41% of cases where resuscitation was attempted, and 30-day survival was 14%. of patients who were transported to hospital with ROSC or ongoing CPR, the survival to discharge was 37%. Of all cases where resuscitation was attempted, 13% showed a favorable neurological outcome (CPC score of 2 or lower) at discharge. The most recent study investigating survival from OHCA in Europe is the EuReCa TWO study [8]. The ROSC rate in this study was calculated at 33%, with 8% being discharged from the hospital alive. Slovenia had an ROSC rate of 37% and survival if treated in hospital of 32% [8]. This shows a 4% increase in ROSC and 5% increase in survival to discharge compared to the EuReCa TWO [8].

The location of the OHCA plays a critical role, influencing the level of care and subsequent outcomes for patients. In Slovenia, the prevalence of AEDs is notable in the vast majority of public buildings, yet residential neighborhoods face challenges with an underdeveloped network of AEDs, marked by poor maintenance and documentation [27]. This scenario raises concerns, particularly considering the increasing number of individuals aged over 65 years living alone [28]. In such cases, the absence of someone present makes it challenging to recognize cardiac arrest, call for help, and initiate CPR.

Given that the majority of OHCA cases occur in homes, it is imperative for policies to consider alternative strategies beyond relying on non-governmental organizations and businesses to place AEDs in public places and buildings, where only 1.7% of CA occur in Slovenia. Some authors argue that the current strategy of placing AEDs in public spaces is a failed approach and advocate for a shift in technology placement towards individual homes [29].

AEDs continue to play a pivotal role in bystander response to OHCA. Our study reveals that the utilization of an AED significantly increases the likelihood of survival when defibrillation occurs. However, when defibrillation does not take place, the use of an AED decreases the odds of a positive outcome compared to no AED use, most probably due to non-shockable rhythm. The observed relationship between AED use and lower survival in cases of a non-shockable rhythm has also been found in the in-hospital setting [30]. Recognizing that the probability of survival significantly improves with rapid defibrillation facilitated by an on-site AED [31], the utilization of AEDs by bystanders remains a critical component of the early response to OHCA.

In our study, AED usage was 19.7%, with a low percentage of shockable rhythms, resulting in only 5.8% of patients receiving a shock. We believe this percentage of AED use is too high, as another recent study in Slovenia found that AED was used by bystanders only once out of 381 OHCAs [32]. The drawback of the Utstein style that it does not track who used the AED, only if the AED was used before EMS arrival. As Slovenia has a first-responder system, our high percentage is probably due to the use of AED by the first responders, who carry AED with them, and not bystanders. Despite the substantial number of AEDs, there exist considerable interregional and intraregional variations, exceeding a factor of 10, which cannot be attributed solely to differences in population density and distribution [33]. Enhancing the rate of AED use could be achieved by moving beyond random placement strategies and instead analyzing population characteristics, OHCA study data, and carefully planning the strategic placement of these expensive devices.

We determined that OHCA was witnessed in 57.1%, which is lower than the European average of 66.6% [8]. Witnessing frequency serves as a significant precursor to bystander response, which was low. Only 33.6% performed CPR with both ventilation and chest compressions, whilst 28.7% performed chest compressions alone. In comparison, the European average is nearly double, at 58.0% [8].

Our regression analysis aimed to assess the impact of bystander CPR on ROSC, survival to discharge, and 30-day survival. Surprisingly, our findings indicated that bystanders had close to zero or slightly negative impact on all these outcome measures. It is crucial to interpret these results cautiously and not conclude that bystanders should refrain from attempting CPR. Instead, we propose that these results highlight untapped bystander potential. Existing research underscores the positive impact of quick bystander response and AED usage on survival outcomes [34], making our results somewhat atypical. We hypothesize that the CPR performed by bystanders in our study may have been of low quality. Several factors could influence the quality of bystander CPR, and one study emphasizes the significance of conventional CPR training for bystanders to enhance its quality [35].

Our study underscores the imperative need for a national CPR education program. Our findings suggest that performing cardiac compressions alone yields superior outcomes when compared to the combination of cardiac compressions with ventilation. A critical turning point in the debate on the need for mechanical ventilation occurred in 1995, when a study conducted on a porcine model of cardiac arrest revealed that positive pressure mechanical ventilation did not enhance resuscitation success or post-resuscitation outcomes [36]. Numerous human studies have since provided evidence supporting the exclusion of mouth-to-mouth ventilation from bystander CPR guidelines [37,38,39]. This is attributed to factors such as an increased likelihood of bystanders initiating CPR, the suboptimal quality of mouth-to-mouth ventilation, and the detrimental effects of prolonged interruptions in chest compressions during ventilation [40]. However, the findings of the EuReCa TWO study strongly advocate for the continued inclusion of teaching chest compressions together with ventilation in bystander CPR education courses [41]. Our results warrant further investigation.

The challenge of educating the general public to remove some of the barriers to provide CPR is global [42]. The proportion of the general population trained in CPR varies significantly around the world (3% to 79%) [42]. Aside from CPR courses integrated into the process of obtaining a driver’s license, Slovenia lacks mandatory CPR education for laymen. In 2010, approximately 70% of Slovenians had previously attended a CPR course, with nearly 80% having done so more than a decade ago. The general knowledge level regarding CPR was evaluated as poor [43].

Certain primary schools collaborate with local organizations to provide CPR and AED lessons [44,45]. These efforts are locally coordinated and are not mandated by national regulations. These CPR trainings could be used as a starting point for a nationwide adoption of CPR and AED courses in elementary schools. The findings indicate the courses contribute significantly to the students’ knowledge enhancement [46,47].

At the turn of the millennium, Denmark implemented an array of solutions to mitigate the negative outcomes associated with OHCA. They introduced mandatory CPR training in elementary schools and as part of acquiring a driver’s license. Additionally, there was encouragement for voluntary CPR courses, distribution of instruction training kits, improvement of dispatch telephone instructions to bystanders, development of an AED network, enhancement of clinical guidelines, provision of additional training to medical personnel, and a reorganization of the emergency medical service (EMS). Over the 10-year period, the proportion of patients receiving bystander CPR increased from 21.1% to 44.9%, the proportion of defibrillated patients doubled from 1.1% to 2.2%, and the 1-year survival rate increased from 2.9% to 10.2% [48].

The Slovenian system has proved effective in providing telephone CPR instructions, as the dispatchers accurately identified OHCA in 67.3% of all cases and provided CPR instructions in 82.0% of those instances. This is well above the European average, measured at 53.2% of OHCAs, and even better than the best-performing country in that dataset, in which dispatchers identified 81.3% of OHCAs in the study [8].

The recent publication of a comprehensive meta-analysis and systematic review, encompassing over 1,081,040 OHCA cases, has shown that community-based interventions, which include education on CPR and AED use, result in a substantial increase in bystander assistance rates during OHCA incidents [49]. This heightened bystander engagement in turn contributes to an improvement in overall survival rates and outcomes of patients [49]. They found that the best results come from combining multiple interventions [49]. Slovenia is currently in the process of revising its school curriculum and is therefore well positioned to adhere to the Internati sonal Liaison Committee on Resuscitation (ILCOR) guidelines of “Kids Save Lives [50]”. As our model does not count bystander interventions as meaningful, it is imperative to start educating lay public.

When interpreting the data collected from registries, it must be noted that registries have their own limitations. The study sample was small and the study length short. The results only show the direction in which further research is warranted. The biggest drawback of the SiOHCA registry was the paper step in the data workflow. The entering of data from the paper into the digital form made the process prone to errors, which are hard to identify without going through the database on a case-by-case basis, which was done for quality control of the SiOHCA database. Timestamps were captured from two different sources: the first was the dispatch service for Slovenia, which has the same clock across all jurisdictions, and the second was the clock of the EMS staff, which then handwrote the timestamp on the paper form. With further digitization of the process and one dispatch center for all the country, these timing shortcomings could be eliminated. Bystander CPR quality was not measured, and there is no method available in Slovenia of measuring it as of the date of writing this article. In future, using video calls could be a possibility to assess the quality of layperson CPR efforts. We also found that answers about AED use were different for different questions (first rhythm and AED use), and this points to a problem with collecting data manually on a separate form. Dataset accuracy could be further improved by implementing Utstein guidelines inside the form used for care of the patients, instead of collecting data separately [15].

## 5. Conclusions

Aligned with the ILCOR statement, the study recommends a change in policy. This change involves the implementation of mandatory CPR and AED training in schools, along with community outreach efforts to educate older generations on how to save an OHCA patient. This is particularly crucial considering that the most common location for an OHCA is at home, and laypersons within the household may be the only available help for the patient. The AED network is currently suboptimally organized. To improve outcomes and evidence-based placement, guidelines should be implemented.

## Figures and Tables

**Table 1:** Utstein template report of the study (AED: automatic external defibrillator, CPR: cardiopulmonary resuscitation, ID CA: identified cardiac arrest, bCPR: CPR with ventilation and chest compressions, CCs: chest compressions, Vent: ventilation, DNAR: do not attempt cardiopulmonary resuscitation document signed, % calculated from total number of cases unless stated otherwise).

Measure	All Cases (n, %)
Population served (n)	1,520,895
Total number of cases	294
Resuscitation attempted	206 (70.0)
Mean age in years (SD)	69.73 (16.67)
Male gender	199 (67.7)
Location of collapse	
At home	203 (69)
At work	1 (0)
Place of sports/recreation	2 (1)
Street/highway	34 (12)
Public building	5 (2)
Residential care/nursing home	16 (5)
Educational institution	0 (0)
Other	32 (11)
Witnessed collapse	
Bystander	168 (57)
EMS	32 (11)
Unwitnessed	92 (31)
Unknown	2 (1)
Bystander response	
Bystander CPR	
no CPR	92 (31)
bCPR	22 (7)
CCs only	127 (43)
CPR but no further details provided	13 (4)
Unknown	40 (13)
AED use before EMS arrival	
Analyze	58 (20)
Shock	17 (6)
First rhythm (% of resuscitation attempted cases)	
Ventricular fibrillation	41 (14)
Ventricular tachycardia	2 (1)
Pulseless electrical activity	43 (15)
Asystole	63 (21)
Bradycardia	2 (1)
AED non-shockable	35 (12)
AED shockable	17 (6)
Not recorded	0 (0)
Etiology	
Cardiac/medical	216 (73)
Trauma	24 (8)
Overdose	2 (1)
Drowning	0 (0)
Electrocution	0 (0)
Asphyxia	12 (4)
Not recorded	40 (14)
EMS Process	
First responder activated	60 (20)
First responder performed CPR	60 (20)
First defibrillation time (mean, mm:ss)	25:16
Drugs given	150 (51)
Hospital Process	
Reperfusion attempted	30 (10)
Organ donation	3 (1)
Targeted temp control	
Indicated—done	24 (8)
Indicated—not done	0 (0)
Not indicated	30 (10)
Unknown	227 (77)
Dispatcher process	
Dispatcher ID CA	
Yes	198 (67)
No	30 (10)
Unknown	66 (22)
Dispatcher CPR	
Yes	241 (82)
No	53 (18)
Unknown	0 (0)
Outcomes (% of those where resuscitation was attempted)	
ROSC	85 (41)
Survival to hospital	
Ongoing CPR	11 (5)
Alive with ROSC	68 (33)
Survival	
To hospital discharge	27 (13)
30 days	30 (14)
Neurological outcomes on discharge	
CPC 1–2	26 (13)
mRS 0–3	17 (8)
Resuscitation not attempted	
DNAR	33 (11)
Obviously dead	55 (19)
Signs of life	0 (0)

**Table 2:** Comparing outcomes when dispatcher gave CPR instructions and when not (CPR: cardiopulmonary resuscitation, EMS: emergency medical service staff, summation of column values in bold).

Bystander Response	Dispatcher Gave CPR Instructions		Dispatcher Did Not Give CPR Instructions	
	EMS Performed CPR (n, %)	EMS Did Not Perform CPR (n, %)	EMS Performed CPR (n, %)	EMS Did Not Perform CPR (n, %)
Chest compressions	105 (51%)	18 (51%)	0	4 (8%)
Chest compressions together with ventilation	21 (10%)	0	0	1 (2%)
No bystander CPR	45 (22%)	13 (37%)	0	34 (64%)
Unknown	35 (17%)	4 (11%)	0	14 (26%)
SUM	206	35	0	53

**Table 3:** R^2^ values predicting outcome when first CPR is carried out by bystander with subgroup characteristics (S: survived, DNS: did not survive, CI: confidence interval 90th percentile).

Outcome Evaluated	R^2^	Mean Age + CI of Those Who Survived until Hospital Discharge	Mean Age + CI of Those Who Did Not Survive	Mean Response Time + CI Those Who Survived until Hospital Discharge	Mean Response Time + CI Those Who Did Not Survive	% of Males in Cohort That Survived until Hospital Discharge	% of Males in Cohort That Did Not Survive
ROSC S = 85 DNS = 121	−0.615886171	66.02 (62.6–69.44)	70.68 (67.93–73.42) N = 121	10 min 43 s (9 min 32.6 s–11 min 55.12 s)	14 min 32 s (12 min 16 s–16 min 48 s)	69.41 (59)	66.27 (169)
30-day survival S = 27 DNS = 255	−0.486323581	58.81 (53.88–63.75)	70.84 (68.82–72.86) N = 255	9 min 41 s (87 min 43.47 s–11 min 44 s)	13 min 20 s (12 min 4 s–14 min 35 s)	85.18 (23)	71.07 (86)
Survival to discharge S = 27 DNS = 254	−0.359089312	58.48 (53.63–63.33)	70.87 (68.85–72.89) N = 254	8 min 56.48 s (7 min 32.31 s–10 min 20.64 s)	13 min 20 s (12 min 4 s–14 min 35 s)	85.18 (23)	66.14 (168)

## Data Availability

The datasets generated during and/or analyzed during the current study are available in the Slovenian Social Science Data Archives repository: Petravić, L., Burger, E., Keše, U., Kulovec, D., Lopes, F., Miklič, R., Pintarič, T., Poljanšek, E., Šircelj, G., Tomšič, G., Turnšek, E., Brezovnik, M. and Strnad, M. (2024). SiOHCA: Out-of-hospital cardiac arrests in Slovenia, 2022 [Data file]. Ljubljana: Univerza v Ljubljani = University of Ljubljana, Arhiv družboslovnih podatkov = Slovenian Social Science Data Archives. ADP—IDNo: SIOHCA22. https://doi.org/10.17898/ADP_SIOHCA22_V1, accessed on 22 February 2024.

## Supplementary Materials

The following supporting information can be downloaded at https://www.mdpi.com/article/10.3390/medicina60081227/s1. Figure S1: The path the patient with OHCA takes in Slovenia; Table S1: Model results for both ROSC and 30-day survival or survival until discharge from hospital; Methods S1: Additional methodology.

## Abbreviations

AED	automated external defibrillator
ALS	advanced life support
bCPR	CPR with ventilation and chest compressions
BLS	basic life support
CCs	chest compressions
CI	confidence interval
CPC	cerebral performance category
DNAR	do not attempt resuscitation
DNS	did not survive
EMS	emergency medical service
EuReCa TWO	European Registry of Cardiac Arrest Two
GDP	gross domestic product
IBM SPSS	International Business Machines Corporation Statistical Package for the Social Sciences
ID CA	identified cardiac arrest
ILCOR	International Liaison Committee on Resuscitation
OHCA	out-of-hospital cardiac arrest
PEA	pulseless electrical activity
ROSC	return of spontaneous circulation
S	survived
SD	standard deviation
STROBE	Strengthening the Reporting of Observational Studies in Epidemiology
Vent	ventilation
VF	ventricular fibrillation

## References

[1] Myat A., Song K.-J., Rea T. (2018). Out-of-Hospital Cardiac Arrest: Current Concepts. Lancet.

[2] Gräsner J.-T., Lefering R., Koster R.W., Masterson S., Böttiger B.W., Herlitz J., Wnent J., Tjelmeland I.B.M., Ortiz F.R., Maurer H. (2016). EuReCa ONE 27 Nations, ONE Europe, ONE Registry: A Prospective One Month Analysis of out-of-Hospital Cardiac Arrest Outcomes in 27 Countries in Europe. Resuscitation.

[3] Lancet T. (2018). Out-of-Hospital Cardiac Arrest: A Unique Medical Emergency. Lancet.

[4] Yan S., Gan Y., Jiang N., Wang R., Chen Y., Luo Z., Zong Q., Chen S., Lv C. (2020). The Global Survival Rate among Adult Out-of-Hospital Cardiac Arrest Patients Who Received Cardiopulmonary Resuscitation: A Systematic Review and Meta-Analysis. Crit. Care.

[5] Oving I., Masterson S., Tjelmeland I.B.M., Jonsson M., Semeraro F., Ringh M., Truhlar A., Cimpoesu D., Folke F., Beesems S.G. (2019). First-Response Treatment after out-of-Hospital Cardiac Arrest: A Survey of Current Practices across 29 Countries in Europe. Scand. J. Trauma. Resusc. Emerg. Med..

[6] Blewer A.L., Ho A.F.W., Shahidah N., White A.E., Pek P.P., Ng Y.Y., Mao D.R., Tiah L., Chia M.Y.-C., Leong B.S.-H. (2020). Impact of Bystander-Focused Public Health Interventions on Cardiopulmonary Resuscitation and Survival: A Cohort Study. Lancet Public Health.

[7] Škufca Strle M., Baznik Š., Skufca Sterle M., Baznik S., Vajd R., Gričar M. (2013). Kako Izboljšati Preživetje Po Kardiopulmonalni Reanimaciji?. Zbornik Prispevkov: Urgentna Medicina: Izbrana Poglavja.

[8] Gräsner J.-T., Wnent J., Herlitz J., Perkins G.D., Lefering R., Tjelmeland I., Koster R.W., Masterson S., Rossell-Ortiz F., Maurer H. (2020). Survival after Out-of-Hospital Cardiac Arrest in Europe—Results of the EuReCa TWO Study. Resuscitation.

[9] Breckwoldt J., Lockey A., Bossaert L., Mistiaen L., Georgiou M. (2013). National Implementation of CPR Training Programmes for School Children in Europe. Resuscitation.

[10] Perkins G.D., Jacobs I.G., Nadkarni V.M., Berg R.A., Bhanji F., Biarent D., Bossaert L.L., Brett S.J., Chamberlain D., de Caen A.R. (2015). Cardiac Arrest and Cardiopulmonary Resuscitation Outcome Reports: Update of the Utstein Resuscitation Registry Templates for Out-of-Hospital Cardiac Arrest: A Statement for Healthcare Professionals from a Task Force of the International Liaison Committee on Resuscitation (American Heart Association, European Resuscitation Council, Australian and New Zealand Council on Resuscitation, Heart and Stroke Foundation of Canada, InterAmerican Heart Foundation, Resuscitation Council of Southern Africa, Resuscitation Council of Asia); and the American Heart Association Emergency Cardiovascular Care Committee and the Council on Cardiopulmonary, Critical Care, Perioperative and Resuscitation. Circulation.

[11] Vandenbroucke J.P., von Elm E., Altman D.G., Gøtzsche P.C., Mulrow C.D., Pocock S.J., Poole C., Schlesselman J.J., Egger M. (2007). STROBE Initiative Strengthening the Reporting of Observational Studies in Epidemiology (STROBE): Explanation and Elaboration. PLoS Med..

[12] Gross Domestic Product by Measures and Year. https://www.stat.si/statweb.

[13] Grmec Š., Križmarič M., Mally Š., Koželj A., Špindler M., Lešnik B. (2007). Utstein Style Analysis of Out-of-Hospital Cardiac Arrest—Bystander CPR and End Expired Carbon Dioxide. Resuscitation.

[14] Fink A., Čander D., Kelebuda D., Gorjup D., Strnad M., Špindler M., Brezovnik M., Rajapakse R., Koren T. (2017). Slovenski Indeks Za Nujno Medicinsko Pomoč.

[15] Petravić L., Burger E., Keše U., Kulovec D., Miklič R., Poljanšek E., Tomšič G., Pintarič T., Lopes M.F., Turnšek E. (2023). How Can Out-of-Hospital Cardiac Arrest (OHCA) Data Collection in Slovenia Be Improved?. Medicina.

[16] Repše J. (2022). Vloga Certificiranih Prvih Posredovalcev v Sistemu Zaščite in Reševanja: Diplomsko Delo = Role of Certified First Responders in Rescue and Protection System. Bachelor’s Thesis.

[17] Perkins G.D., Gräsner J.-T., Semeraro F., Olasveengen T., Soar J., Lott C., Van De Voorde P., Madar J., Zideman D., Mentzelopoulos S. (2021). European Resuscitation Council Guidelines 2021: Executive Summary. Resuscitation.

[18] Petravić L., Burger E., Kulovec D., Lopes M.F., Pintarič T., Poljanšek E., Šircelj G., Tomšič G., Turnšek E., Brezovnik M. SiOHCA: Out-of-Hospital Cardiac Arrests in Slovenia, 2022; Data File; 2024. https://www.adp.fdv.uni-lj.si/opisi/siohca22/.

[19] Tenny S., Boktor S.W. (2023). Incidence. StatPearls.

[20] Burger E. (2024). Untapped Bystander Potential in Slovenia: Out-of-Hospital Cardiac Arrests Utstein Style Report—Data Analysis. https://zenodo.org/records/10660023.

[21] (2023). SURS Slovenian Population in 2023. Slovenian Census Data from 2023. https://www.stat.si/StatWeb/en.

[22] Tadel S., Horvat M., Noc M. (1998). Treatment of Out-of-Hospital Cardiac Arrest in Ljubljana: Outcome Report According to the ‘Utstein’ Style. Resuscitation.

[23] Randjelovic S.S., Nikolovski S.S., Tijanic J.Z., Obradovic I.A., Fiser Z.Z., Lazic A.D., Raffay V.I. (2023). Out-of-Hospital Cardiac Arrest Prospective Epidemiology Monitoring during the First Five Years of EuReCa Program Implementation in Serbia. Prehosp. Disaster Med..

[24] Važanić D. (2022). Out-of-Hospital Cardiac Arrest Outcomes—Bystander Cardiopulmonary Resuscitation Rate Improvement. Acta Clin. Croat..

[25] Zalihić A., Šljivo A., Ribić E., Gavranović A., Brigić L. (2022). Bystanders’ Cardiopulmonary Resuscitation Involvement in the Treatment of out-of-Hospital Cardiac Arrest Events and Educational Status Regarding Basic Life Support Measures and Automated External Defibrillator Usage among Residents in Canton Sarajevo, Bosnia and Herzegovina. Med. Glas. Ljek. Komore Zenicko-Doboj. Kantona.

[26] Zaletel M., Vardič D., Hladnik M. (2024). Zdravstveni Statistični Letopis Slovenije 2022.

[27] Ručigaj S., Demšar L. (2018). AED Baza Slovenije: Stanje Evidence Javno Dostopnih Naprav AED in Ažurnost Informacij o Napravah Vpisanih v Bazo = A Follow-up on Slovenian Public-Access AEDs Database. Urgentna Medicina: Izbrana Poglavja 2018.

[28] (2022). Mateja, Kotnik Veliko Mladih Živi Skupaj s Starši, Čedalje več Starejših Prebiva Samih. Delo.

[29] Brooks S.C. (2024). Public Access Defibrillation Is a Failed Strategy. Can. J. Emerg. Med..

[30] Chan P.S., Krumholz H.M., Spertus J.A., Jones P.G., Cram P., Berg R.A., Peberdy M.A., Nadkarni V., Mancini M.E., Nallamothu B.K. (2010). Automated External Defibrillators and Survival After In-Hospital Cardiac Arrest. JAMA.

[31] Elhussain M.O., Ahmed F.K., Mustafa N.M., Mohammed D.O., Mahgoub I.M., Alnaeim N.A., Ali R., Bushra N., Ahamed H.K., Abdelrahman N. (2023). The Role of Automated External Defibrillator Use in the Out-of-Hospital Cardiac Arrest Survival Rate and Outcome: A Systematic Review. Cureus.

[32] Strnad M., Borovnik Lesjak V., Jerot P., Esih M. (2023). Prehospital Predictors of Survival in Patients with Out-of-Hospital Cardiac Arrest. Medicina.

[33] Rucigaj S., Podobnik B., Gradisek P., Sostaric M. (2019). “AED Database of Slovenia”—An Analysis of Operation of Slovenian National Public Access Defibrillators Registry. Resuscitation.

[34] Rao P., Kern K.B. (2018). Improving Community Survival Rates from Out-of-Hospital Cardiac Arrest. Curr. Cardiol. Rev..

[35] Chocron R., Jobe J., Guan S., Kim M., Shigemura M., Fahrenbruch C., Rea T. (2021). Bystander Cardiopulmonary Resuscitation Quality: Potential for Improvements in Cardiac Arrest Resuscitation. J. Am. Heart Assoc..

[36] Noc M., Weil M.H., Tang W., Turner T., Fukui M. (1995). Mechanical Ventilation May Not Be Essential for Initial Cardiopulmonary Resuscitation. Chest.

[37] Hallstrom A., Cobb L., Johnson E., Copass M. (2000). Cardiopulmonary Resuscitation by Chest Compression Alone or with Mouth-to-Mouth Ventilation. N. Engl. J. Med..

[38] Virkkunen I., Kujala S., Ryynänen S., Vuori A., Pettilä V., Yli-Hankala A., Silfvast T. (2006). Bystander Mouth-to-Mouth Ventilation and Regurgitation during Cardiopulmonary Resuscitation. J. Intern. Med..

[39] SOS-KANTO Study Group (2007). Cardiopulmonary Resuscitation by Bystanders with Chest Compression Only (SOS-KANTO): An Observational Study. Lancet.

[40] Cabrini L., Biondi-Zoccai G., Landoni G., Greco M., Vinciguerra F., Greco T., Ruggeri L., Sayeg J., Zangrillo A. (2010). Bystander-Initiated Chest Compression-Only CPR Is Better than Standard CPR in out-of-Hospital Cardiac Arrest. HSR Proc. Intensive Care Cardiovasc. Anesth..

[41] Pourghaderi A.R., Kogtikov N., Lees M.H., Cai W., Pin Pek P., Fu Wah Ho A., Ming Ng W., Kwak J., Elgin White A., Lynn Lim S. (2022). Maximum Expected Survival Rate Model for Public Access Defibrillator Placement. Resuscitation.

[42] Birkun A., Gautam A., Trunkwala F. (2021). Global Prevalence of Cardiopulmonary Resuscitation Training among the General Public: A Scoping Review. Clin. Exp. Emerg. Med..

[43] Rajapakse R., Noč M., Kersnik J. (2010). Public Knowledge of Cardiopulmonary Resuscitation in Republic of Slovenia. Wien. Klin. Wochenschr..

[44] Generacija Rešuje Življenja|ZD Litija. https://zd-litija.si/generacija-resuje-zivljenja/.

[45] Osnovnošolske Ekipe Prve Pomoči—Rdeči Križ Slovenije—Območno Združenje Ljubljana. https://www.rdecikrizljubljana.si/sl/Osnovnosolske_ekipe_prve_pomoci/.

[46] Vajd R., Gričar M., Prestor J., Bračko V. (2017). Mednarodni Simpozij o Urgentni Medicini, 24 Urgentna Medicina: Izbrana Poglavja 2017: Zbornik = International Symposium of Emergency Medicine, 24 Emergency Medicine: Choosen Topics 2017.

[47] Vajd R., Zelinka M. (2023). Mednarodni Simpozij o Urgentni Medicini, 29 Urgentna Medicina = International Symposium of Emergency Medicine, 29 Emergency Medicine: Choosen Topics 2023.

[48] Wissenberg M., Lippert F.K., Folke F., Weeke P., Hansen C.M., Christensen E.F., Jans H., Hansen P.A., Lang-Jensen T., Olesen J.B. (2013). Association of National Initiatives to Improve Cardiac Arrest Management with Rates of Bystander Intervention and Patient Survival After Out-of-Hospital Cardiac Arrest. JAMA.

[49] Simmons K.M., McIsaac S.M., Ohle R. (2023). Impact of Community-Based Interventions on out-of-Hospital Cardiac Arrest Outcomes: A Systematic Review and Meta-Analysis. Sci. Rep..

[50] Schroeder D.C., Semeraro F., Greif R., Bray J., Morley P., Parr M., Kondo Nakagawa N., Iwami T., Finke S.-R., Malta Hansen C. (2023). KIDS SAVE LIVES: Basic Life Support Education for Schoolchildren: A Narrative Review and Scientific Statement from the International Liaison Committee on Resuscitation. Circulation.

