# Supplementary material for: Enhancing Bystander Intervention: Insights from the Utstein Analysis of Out-of-Hospital Cardiac Arrests in Slovenia
Source: Medicina (Kaunas). 2024 Jul 29;60(8):1227. doi: 10.3390/medicina60081227 (PMC11356526; doi:10.3390/medicina60081227)
Supplement: Supplementary file 1 [file medicina-60-01227-s001.zip › Supplementary methods .pdf]

Methods Supplement

Data pre-processing

Data preprocessing was as minimal as possible. Missing values for categorical features were encoded as unknown as this is already an option for each variable. In case of numerical variables there were missing values for response times and defibrillation times.

In the case of missing values in defibrillation times there were missing values in almost 65 % of cases due to the fact that data on defibrillation time exists only if defibrillation actually happened and not otherwise. We encoded the defibrillation times as bins of similar sizes and created an indicator variable for each bin as well as a separate indicator variable in the case where defibrillation did not happen.

There were 7.3 % of missing values in the case of response times. We explored mean and median imputation which were passed to the models as a parameter.

The dataset consists mostly of categorical variables which were encoded as integers and further one-hot-encoded to avoid being treated as ordered values. Reference categories were chosen and removed manually for easier interpretation.

Numerical variables which were patient age and response time were imputed if necessary and standardized for each fold when tuning the models.

In the dataset two distinct values are used in case of unknown answer – unknown or not recorded. There is a technical difference between the two variables where unknown means that an attempt was made to answer the question but the data was not found, whereas not recorded means that the data was not found and no attempt was made to find it. For the purposes of the analysis both unknown and not recorded answers were encoded as unknown.

A limitation to this is also the fact that some variables that are not applicable are also encoded as unknown. For example the variable describing bystander AED use is only applicable when there is a bystander, when there is no bystander it's encoded as unknown.

Target variables were ROSC and 30 day survival or survival untill discharge. Both were encoded as indicators. Models were developed separately for each target variable.

Model description

Due to multicollinearity in our dataset straightforward approach in the development of the logistic regression model was not possible so we introduced regularization where L1 and L2 penalties were explored. The chosen regularization parameter minimized the mean log score value of the model after leave one out cross validation.

We built each forest with 5000 trees. Number of variables randomly sampled as candidates at each split was set to 2 (for ROSC) or square root of the number of features (for survival) and minimum size of terminal nodes was set to 1 after evaluating the model's mean log score after leave one out cross validation. We used median imputation.

We estimated feature importances by visualising the variability of mean decrease accuracy and mean decrease gini after cross validation with the help of boxplots.

The analysis was done using Python programming language (version 3.10.4) and several open-source libraries, especially NumPy (version 1.24.1) and Pandas (version 1.4.2). We implemented ridge regression model and evaluation metrics from the scikit-learn library (version 1.1.1) for our analysis. For visualisations we used Matplotlib (version 3.8.2). Implementation of random forest was done in R due to a high number of categorical variables. The randomForest package (version 4.7.1.1) was used for building and evaluating the random forest and varImp (version 0.4) package was used for feature importance analysis. For easier data manipulation we used dplyr (version 1.0.10) and openxlsx (version 4.2.5.2) packages.

Gini of models created

ROSC

After building the random forest model we plotted the variability of mean decrease accuracy and mean decrease gini (Figure S2) after performing leave one out cross validation in order to estimate feature importance.

Mean decrease accuracy measures (Figure S3) the decrease in model accuracy when a specific feature is removed. A higher mean decrease accuracy indicates that the model relies heavily on that feature for making accurate predictions. Higher mean decrease gini values suggest that the feature is more important for making effective splits in the decision trees of the random forest.

From our visualizations, it is evident that variables describing TTM and coronary reperfusion exhibit the highest importance, alongside the variable indicating whether defibrillation occurred or not.

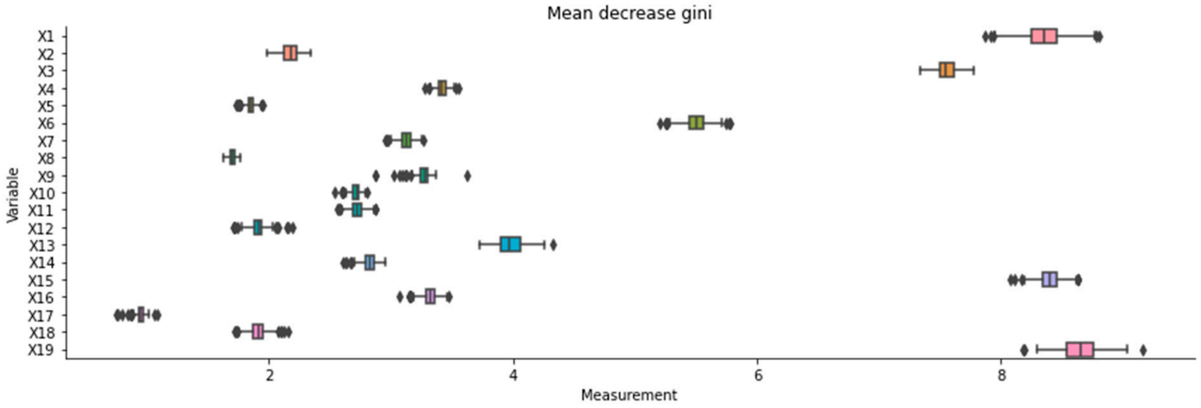

Figure S2: Mean decrease gini for ROSC model

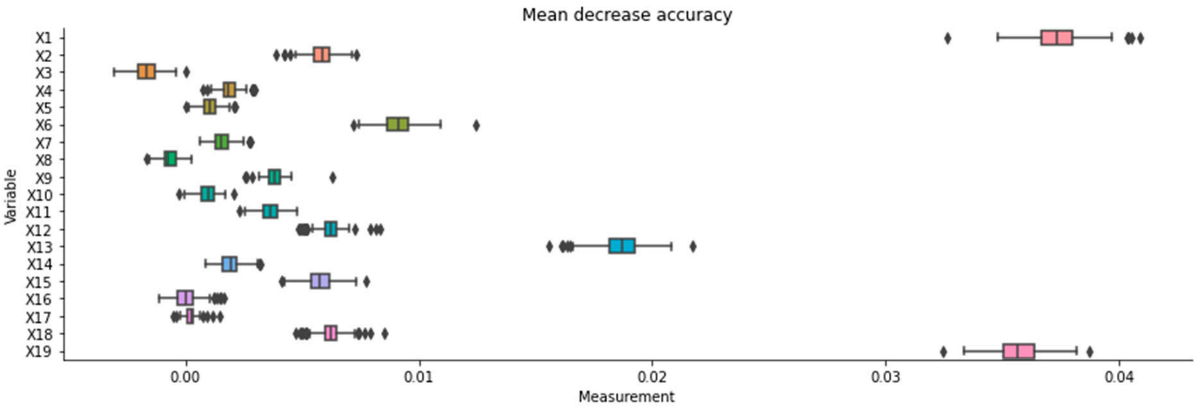

Figure S3: Mean decrease accuracy for ROSC model

30 days survival or survival until hospital discharge model

We observed that features with the highest mean decrease gini (Figure S4) and mean decrease accuracy (Figure S5) values were associated with patient age, the aetiology of arrest, response time, and the location of the event.

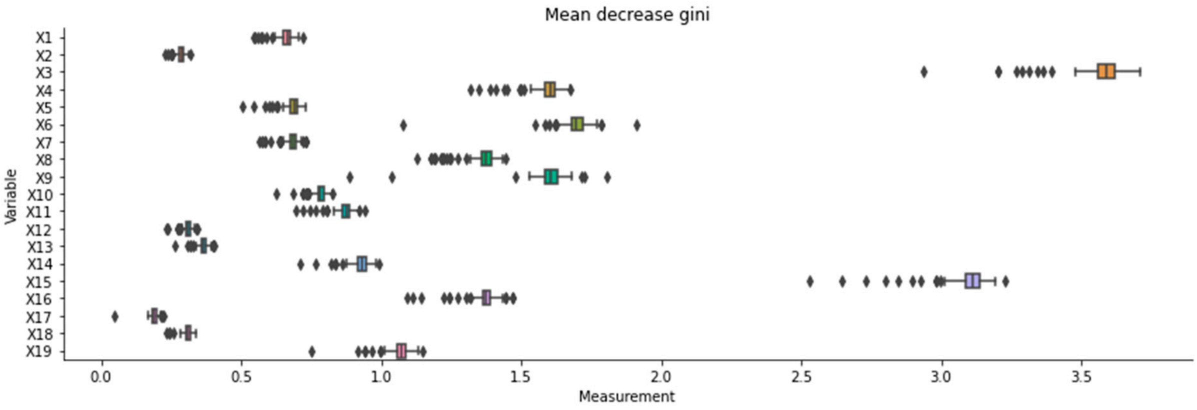

Figure S4: Mean decrease gini for 30 days survival or survival until hospital discharge model

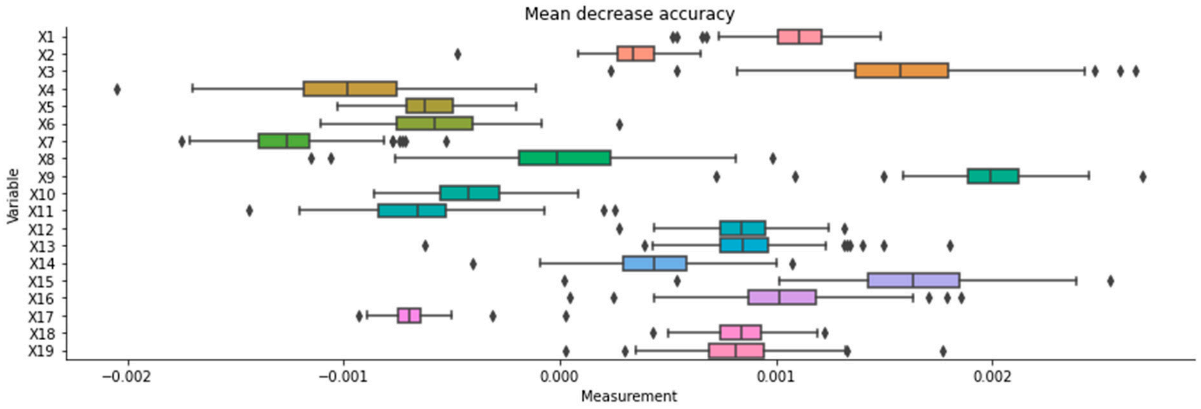

Figure S5: Mean decrease accuracy for 30 days survival or survival until hospital discharge model
