# Supplementary material for: Enhancing Bystander Intervention: Insights from the Utstein Analysis of Out-of-Hospital Cardiac Arrests in Slovenia
Source: Medicina (Kaunas). 2024 Jul 29;60(8):1227. doi: 10.3390/medicina60081227 (PMC11356526; doi:10.3390/medicina60081227)
Supplement: Supplementary file 1 [file medicina-60-01227-s001.zip › Supplementary table 1.pdf]

Table S1: Model results for both ROSC and 30 days survival or survival until discharge from hospital models

Variable and answer	Mean posterior odds ratio and 95 % confidence interval for ROSC	Mean posterior odds ratio and 95 % confidence interval for 30 days survival or survival until discharge from hospital
Covered by new dispatch_yes	0.96 (0.95 - 0.97)	0.96 (0.95 - 0.97)
Witnessed arrest_EMS witnessed	1.22 (1.21 - 1.23)	1.36 (1.36 - 1.38)
Witnessed arrest_Bystander witnessed	1.14 (1.13 - 1.15)	0.98 (0.97 - 0.99)
Witnessed arrest_Unknown	0.96 (0.96 - 0.96)	0.99 (0.99 - 0.99)
Defibrilation under 10 min	1.25 (1.23 - 1.26)	1.13 (1.12 - 1.14)
Defibrilation over 20 min	1.12 (1.12 - 1.13)	1.15 (1.15 - 1.16)
Defibrilation within 10 - 15 min	1.25 (1.23 - 1.26)	1.13 (1.12 - 1.14)
Defibrilation given in 15 - 20 min	1.06 (1.05 - 1.07)	1.84 (1.82 - 1.84)
No defibrilation	0.66 (0.66 - 0.67)	0.51 (0.51 - 0.51)
Dispatcher identified presence of cardiac arrest_Yes	0.8 (0.79 - 0.81)	0.85 (0.85 - 0.86)
Dispatcher identified presence of cardiac arrest_No	1.13 (1.13 - 1.14)	0.98 (0.97 - 0.99)

Dispatcher identified presence of cardiac arrest_Unknown	1.11 (1.11 - 1.12)	1.2 (1.19 - 1.21)
Coronary reperfusion_Unknown	0.45 (0.44 - 0.45)	0.48 (0.47 - 0.48)
Coronary reperfusion_PCI	1.15 (1.14 - 1.16)	2.36 (2.32 - 2.39)
Coronary reperfusion_Angiography only	1.2 (1.2 - 1.21)	1.28 (1.26 - 1.3)
Arrest location_workplace	0.94 (0.94 - 0.94)	0.96 (0.96 - 0.97)
Arrest location_other	1.06 (1.05 - 1.07)	1.43 (1.43 - 1.45)
Arrest location_public building	1.01 (1.0 - 1.01)	1.42 (1.4 - 1.45)
Arrest location_sports/recreation event	1.11 (1.11 - 1.12)	0.92 (0.92 - 0.92)
Arrest location_ street/highway	1.04 (1.03 - 1.05)	0.61 (0.6 - 0.61)
Arrest location_assisted living/nursing home	1.09 (1.08 - 1.09)	1.19 (1.17 - 1.21)
Bystander response_unknown	1.05 (1.04 - 1.06)	1.39 (1.38 - 1.42)
Bystander response_CPR compression only	0.94 (0.93 - 0.95)	1.22 (1.21 - 1.23)
Bystander response_compression and ventilations	1.0 (0.99 - 1.01)	0.81 (0.8 - 0.82)
Response time	1.0 (1.0 - 1.0)	1.0 (1.0 - 1.0)
Gender_male	0.86 (0.85 - 0.87)	1.31 (1.3 - 1.32)
Age	0.99 (0.99 - 0.99)	0.96 (0.96 - 0.96)
Targeted temperature management_unknwon	0.55 (0.55 - 0.56)	0.33 (0.33 - 0.34)

Targeted temperature management_post-ROSC in-hospital	1.26 (1.25 - 1.27)	1.16 (1.15 - 1.17)
Targeted temperature management_post-ROSC prehospital	1.13 (1.12 - 1.13)	1.28 (1.27 - 1.3)
Bystander AED use_AED used, shock delivered	1.25 (1.23 - 1.26)	1.26 (1.25 - 1.27)
Bystander AED use_AED used, no shock delivered	0.9 (0.9 - 0.91)	0.88 (0.88 - 0.89)
Bystander AED use_unknown	1.08 (1.07 - 1.09)	1.17 (1.16 - 1.19)
Pathogenesis_unknown	0.84 (0.83 - 0.84)	0.82 (0.81 - 0.83)
Pathogenesis_traumatic cause	0.97 (0.96 - 0.97)	0.82 (0.82 - 0.83)
Pathogenesis_asphyxial	1.25 (1.25 - 1.26)	0.92 (0.92 - 0.93)
Pathogenesis_drug overdose	1.03 (1.03 - 1.03)	1.15 (1.14 - 1.15)
First monitored rhythm_AED shockable	1.26 (1.25 - 1.26)	1.26 (1.25 - 1.27)
First monitored rhythm_AED - non-schockable rhythm	1.07 (1.06 - 1.08)	0.87 (0.87 - 0.87)
First monitored rhythm_bradycardia	1.0 (1.0 - 1.0)	1.11 (1.09 - 1.11)
First monitored rhythm_unknown	1.05 (1.05 - 1.06)	1.23 (1.22 - 1.26)
First monitored rhythm_PEA	0.86 (0.85 - 0.87)	0.73 (0.72 - 0.73)
First monitored rhythm_VF	1.25 (1.23 - 1.25)	1.54 (1.52 - 1.55)
First monitored rhythm_pulseless VT	1.12 (1.12 - 1.13)	0.96 (0.96 - 0.96)
Drugs given_Adrenaline	0.88 (0.87 - 0.89)	0.51 (0.5 - 0.51)

Drugs given_Adrenalin and Amiodarone	0.97 (0.97 - 0.98)	0.79 (0.79 - 0.81)
Drugs given_amiodarone	1.03 (1.03 - 1.03)	1.15 (1.14 - 1.15)
Drugs given_unknown	1.05 (1.04 - 1.05)	0.95 (0.95 - 0.96)
