# Supplementary material for: Enhancing Bystander Intervention: Insights from the Utstein Analysis of Out-of-Hospital Cardiac Arrests in Slovenia
Source: Medicina (Kaunas). 2024 Jul 29;60(8):1227. doi: 10.3390/medicina60081227 (PMC11356526; doi:10.3390/medicina60081227)
Supplement: Supplementary file 1 [file Supplementary figure 1.pdf › Supplementary figure 1.pdf]

Figure S1: The path the patient with OHCA takes in Slovenia (OHCA: out-of-hospital cardiac arrest, ReNC: regional notification center, EMS: emergency medical services,BLS: basic life support, ALS: advanced life support, ER: emergency room, ICU:intensive care unit).

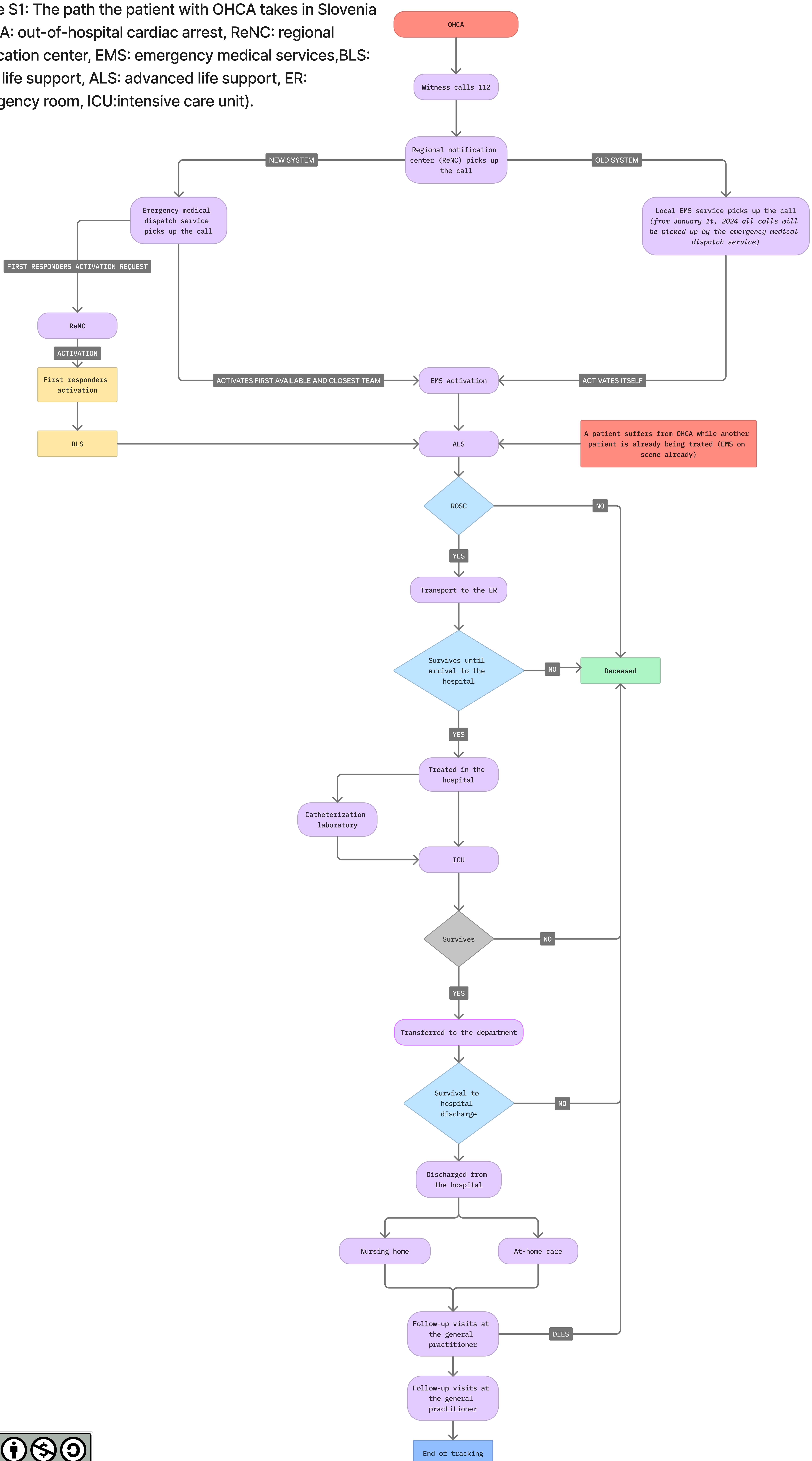

CC BY NC SA
